# Evaluating Nodes of Latent Mediators in Heterogeneous Communities

**DOI:** 10.1038/s41598-020-64548-6

**Published:** 2020-05-21

**Authors:** Hiroko Yamano, Kimitaka Asatani, Ichiro Sakata

**Affiliations:** 1 Institute for Future Initiatives, The University of Tokyo, 7-3-1 Hongo, Bunkyo-ku, Tokyo, 113-0033 Japan; 2 Innovation Policy Research Center, Institute of Engineering Innovation, School of Engineering, The University of Tokyo, 7-3-1 Hongo, Bunkyo-ku, Tokyo, 113-0033 Japan

**Keywords:** Computational science, Information technology, Scientific data

## Abstract

Conventionally, the importance of nodes in a network has been debated from the viewpoint of the amount of information received by the nodes and its neighbors. While node evaluation based on the adjacency relationship mainly uses local proximity information, the community structure that characterizes the network has hardly been considered. In this study, we propose a new node index that contributes to the understanding of the inter-community structure of a network by combining the measures of link distribution and community relevance. The visualization of node rankings and rank correlations with respect to the attack tolerance of networks demonstrated that the proposed index shows the highest performance in comparison with five previously proposed indexes, suggesting a new way to detect latent mediators in heterogeneous networks.

## Introduction

Knowledge heterogeneity has been investigated based on the observation of the benefits of integrating distant knowledge in the heterogeneity of firm collaborations^1–3^ or organizational turnover under environment turbulence^4,5^. Many researchers have demonstrated the effectiveness of incorporating knowledge from rare links with widely accepted concepts such as shortcuts in *small world*^6^, bridges between cliques as *weak ties*^7^, and bridges over *structural holes*^8^. However, contrary to the prevailing conceptual works and case studies, there are fewer studies on the measurement of rarity of the links in a network.

The driving hypothesis of the present study is that the importance of a node is estimated from the heterogeneity of the links it brings. We already know that hubs, which are nodes with many links, are important^9^, but there is comparatively less evidence for the composition or values of the links that make a node important. Most conventional network indexes tend to be affected by the link density of adjacent nodes, such as betweenness centrality, which counts the number of shortest paths via a node^10^; Katz centrality, which takes into account the total number of paths between a pair of nodes^11^; and PageRank, which considers the number of backlinks with their respective importance^12^. These indexes are effective to extract apparently significant nodes that have many important links and a great influence on networks^9^. However, another method is required to find rare nodes that have a few important links.

In the small world network, the shortcuts made with a certain rate of rewiring enables its properties between complete and random graphs: the short average shortest path length and the large average cluster coefficient, which occur simultaneously^6^. The shortcut can be considered to correspond to weak ties. Weak ties were proposed based on the observation that people tended to get useful career information more from individuals outside their community, with whom they have rare contact opportunities, than from people within their community, whom they meet often^7^. The measure of weakness here is the contact frequency, which corresponds to the weight of links in the network, whereas the measure to grasp structural holes uses network topology, known as Burt’s constraint, which represents the absence of a structural hole^13^. Burt’s constraint counts the node degree and the number of common neighbors of every node pair in the network, reflecting local information up to one or two paths from the target node. It was designed to evaluate nodes connecting communities by assuming their potential to provide rare information and to bring about innovation. However, as with the conventional node indexes previously described, this index does not incorporate the characteristics of communities holding nodes beyond three paths away from the target node (for details and the definition, see Methods).

Under the explosion of available network data, researchers have investigated the roles of nodes, offering methods to reduce data complexity and extract meaningful information related to structural dimensions of the network^14–17^. Blockmodel is one such abstraction technique that helps understand the roles of nodes in networks. Assuming that two nodes are structurally equivalent if they are connected to the same nodes, it collapses nodes into blocks based on a given partitioning of the node set^14^. Although blocks represent the overall structure of the network, the model is not enough to clarify how nodes function in heterogeneous communities. Revealing the cases that blockmodel fails to capture the roles of nodes, Guimera *et al*. proposed another way to extract the roles of nodes by incorporating the modular structure and the patterns of link distribution of the networks^15^. He proposed two node indexes: the within-cluster degree Z and participation coefficient P^15^ (see Eqs. (1) (6) in Methods), which clarify the role of each node from the perspectives of how “well connected” and “well distributed” a node is; these perspectives define how a node is positioned in its own cluster (hubs) and between clusters (connectors). Several studies have demonstrated the discriminating power of the indexes, such as the connector firms bridging different regional clusters^18^, emerging research fronts in citation networks^19^, and the role-to-role connectivity^20^. However, by definition, the participation coefficient P only evaluates the link distribution to communities, which does not distinguish the distances between communities. In other words, it is insensible for rare links from the nodes belonging to distant communities, and numerous studies have revealed their qualitative importance and structural values in the network^1–3,6,7,13^.

Within-community degree and its participation coefficient might be too strong a requirement for the analyses of large complex networks. Recent studies have revealed that more detailed community structure contributes to the prediction of missing links^21–24^, in addition to node similarities based on the number of common neighbors in general^25–27^. The first successful concept is based on the hypothesis that node pairs in the same community have higher similarity and, therefore, higher link probability than those in different communities^21^. In contrast to the primary method that only considers direct links within a community, Ding *et al*. used the relationship between communities, called community relevance, for link prediction^24^. By using community relevance, which measures the probability of two communities sharing common neighbors, he achieved better prediction accuracy compared to the existing approaches based on local node similarities.

In this paper, we propose an analyzing schema to comprehend the inter-community structure by combining existing theories of nodal importance and community relevance. We demonstrate that the proposed index shows better performance compared to the participation coefficient P (P) in detecting nodes that connect distant communities. We validate the performance of the proposed index with the visualization of node rankings in networks with varied communities, changes in the network diameter after the removal of top-ranked nodes, and rank correlations with standard ranking, which identifies nodes that would make the average shortest path longer if they are removed. Our approach sheds new light on node values by offering a way to detect latent mediators in heterogeneous communities with different number and density of nodes and links, that is consistent with theories and numerous empirical studies in social and industrial networks^6–8,13,28^.

## Results

### Node evaluation by connectivity and heterogeneity

The roles of nodes have been estimated by their positions in the network, as characterized by the structure of its clusters. The positions, which are related to the connectivity of the nodes, are measured with the index P^15^, which quantifies how “well distributed” the links of node i are among different clusters, given by

1 $${P}_{i}=1-\mathop{\sum }\limits_{s=1}^{{N}_{m}}{\left(\frac{{K}_{is}}{{k}_{i}}\right)}^{2},$$

where ${K}_{is}$ is the number of links from node $i$ to other nodes in community $s$ and ${k}_{i}$ is the total degree of node i (the number of links connected to node i). ${P}_{i}$ is close to 1 if its links are uniformly distributed among all the communities and is 0 if all its links are within its own community. However, there may be cases where it provides a poor representation of the node values in the network, e.g., when the network contains heterogeneous local structures of communities. In such a heterogeneous network, the relevance between communities is significant for assessing the roles of the nodes in addition to their belongings, considering the novelty of information they mediate. For example, consider a class reunion, in which we enjoy talking with old friends not only because we spent time together in our younger days but also because they belong to distant communities that are apart from the ones we belong to. ${P}_{i}$ cannot count such distances because it evenly surveys communities and only measures if a node has within or outer community links. To overcome this limitation, we used an experimentally verified community relevance index called the community relevance Jaccard coefficient (CRJC)^24^ as the weight of ${P}_{i}$. The weighting scheme we use is the negative log average of CRJC; the probability of the two communities that have common neighbors evaluates the rarity of the links connecting distant communities. We named this new index as weighted P (PW), which is defined as follows:

2 $$P{W}_{i}=-\,{P}_{i}\,\log \,\sum _{j\in \Gamma {(i)}^{IC},j\ne i}\,\frac{{\rm{C}}{\rm{R}}{\rm{J}}{\rm{C}}({c}_{i},{c}_{j})}{L+\delta },$$

where ${P}_{i}$ is the index P defined by Eq. (1) and CRJC is the community relevance defined by Eq. (7). Γ${(i)}^{IC}$ is the set of neighbors of node $i$ that do not belong to the cluster of node $i$. $L$ represents the number of the nodes in Γ${(i)}^{IC}$. Delta has an infinitesimal value of 0.000001 to prevent zero division error. $P{W}_{i}$ increases as nodes become connected to more communities with low community relevance. $P{W}_{i}$ counts the novelty of information regarding linkage probability concerning the distances among communities, while ${P}_{i}$ quantifies the overall connectivity of a node to multiple communities.

Figure 2 shows an outline of the calculation procedure with example numbers in matrices and the corresponding network images. Every matrix obtained in the calculation process has its elements arranged in the order of the nodes in the graph. The procedure of obtaining each matrix is briefly described in the following.

#### Adjacent community matrix

We acquired the community IDs of all nodes in the network using the conventional clustering algorithm of the Louvain method^29^. Subsequently, by updating the adjacency matrix of the network, we created a matrix containing the community IDs of adjacent nodes at the position where links existed.

#### Adjacent inter-community relevance matrix

As we focused on the links between communities, we replaced all elements that correspond to within-community links in the adjacent community matrix with 0. Subsequently, we updated the remaining non-zero community IDs with the degrees of community relevance. The weight of a node is obtained by computing the negative logarithm of the average of values represented in the corresponding row of the adjacent inter-community relevance matrix.

### Finding connectors of ego networks

Our first focus was to determine if the new index could distinguish differences in community structures. We use Facebook social data from Stanford Large Network Dataset Collection (SNAP)^30^, which contains ego networks of 10 anonymized users that consist of the users’ friends lists^31^. To evaluate the discrimination capacity of the proposed index, we combined the ego networks and compared the rankings of the nodes by the indexes P and PW. As most of the Facebook users had relatively few common friends, the combined network had few links among the constituent ego networks, which were clustered by the egos. We found some common patterns of PW that occurred in many of the networks, such as a tendency to identify rare friends who connect the ego users or groups in the networks.

**Figure 1:**
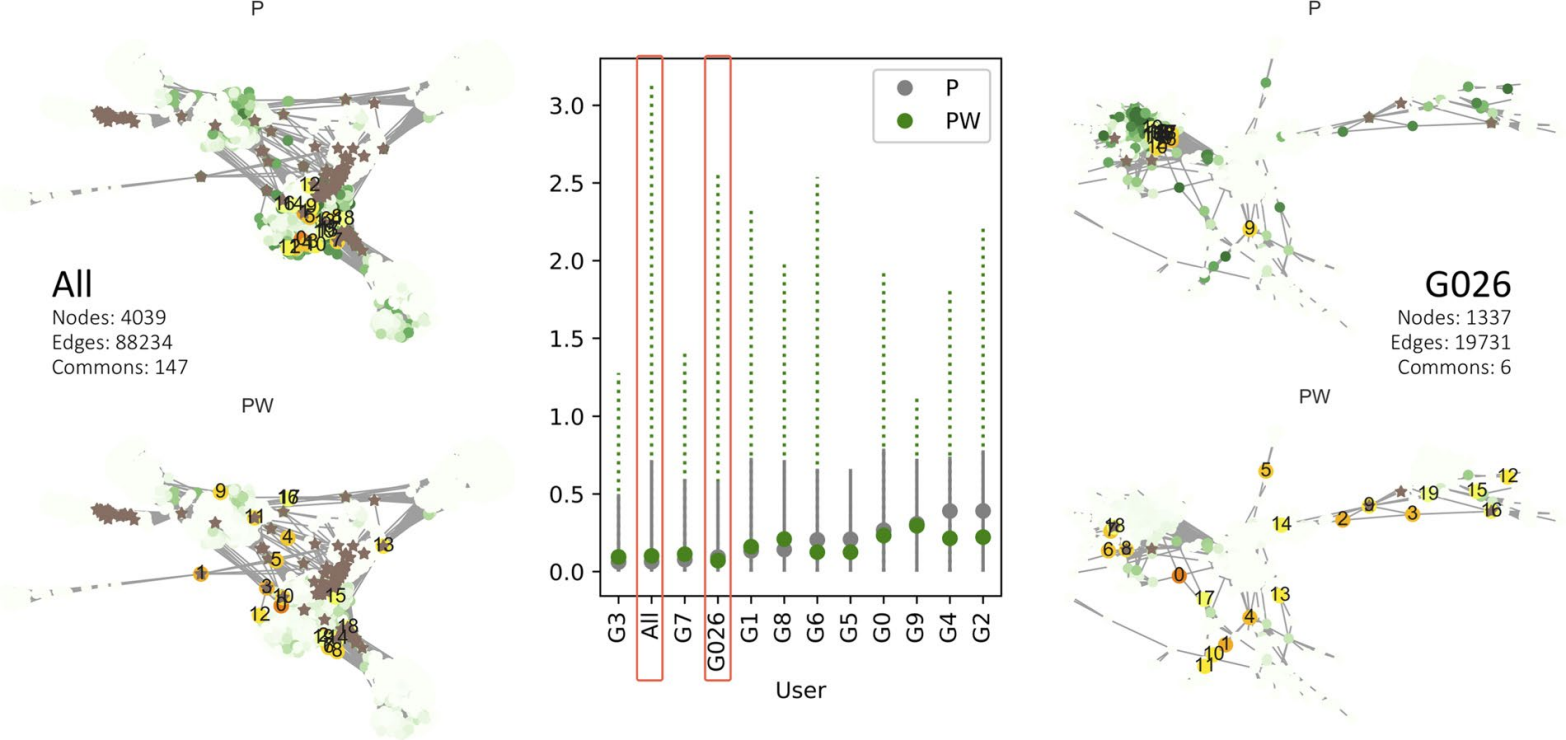
Comparison of the discriminating capacity of P and PW. The bar plot represents the ranges of P and PW for each of 10 Facebook users (G0–G9) and two combined networks of all 10 users and three users (G0, G2, and G6). The mean values of P and PW are represented by grey and green dots, respectively, with bars of these colors representing the range from the minimum to maximum values of the corresponding index (for detailed distributions of P and PW, see Supplementary Fig. S3). The numbers of nodes, edges, and common friends in each combined network are described in the figure. The star-shaped blown nodes in the networks represent common friends between ego users. Top 20 nodes are marked with orange gradation values with labels of the node ranking in each network and the other nodes are colored in green gradations. The thickness of orange and green represents the corresponding values of the nodes in each network.

Figure 1 shows the distributions of the variables for each of the 10 users with two combined networks of all users and three users. In every network, PW had a wider range of the values than P, suggesting that it has a higher capacity to discriminate nodes. The surrounding networks show details for the combined ego users and their common friends with the rankings of the top 20 nodes by P and PW, respectively. All the networks consist of clusters with varied distances, and the common friends lie between the clusters (for visualization of the users and the clusters in the networks, see Supplementary Figs. S1 and S2). Closer inspection of the four networks reveals that the nodes associated with higher PW values showed better performance in detecting common friends than those associated with higher P values, which are accumulated in one part of the network. The values of the top 20 nodes can be found in Supplementary Table S1. We also found that PW tended to detect dispersed common friends, rather than those who were accumulated between close communities.

**Figure 2:**
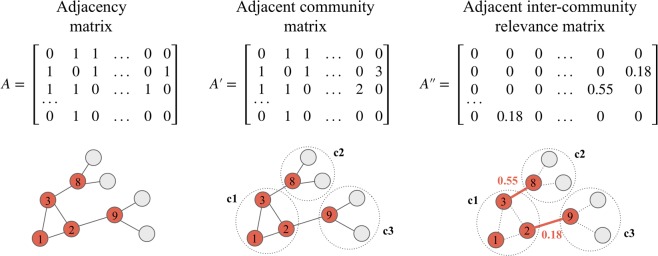
Outline of updating elements in the adjacency matrix to community relevance. The red nodes in the networks are the nodes represented in the matrices. The number of nodes in the network represents the corresponding row indexes in the matrix. Community IDs in the networks are represented as c1, c2, and c3, which correspond to the numbers 1, 2, and 3, respectively, in the adjacent community matrix. The example values of community relevance are represented by the red numbers 0.55 and 0.18 in the right graph, which are equivalent to the values in the adjacent outer community relevance matrix.

### Community relevance

To determine the relations between the proposed index and network structures in detail, we prepared two benchmark networks called the relaxed caveman (RC) network^6^ and Lancichinetti-Fortunato-Radicchi (LFR) network^32^ as well as one real network of inter-firm transactions in Tohoku region (see data in Methods). The degrees of community relevance (CRJC, see Eq. (7) in Methods) of the three networks are listed in Tables 1, 2 and 3. The distributions of the values of community relevance of these networks were characterized by the following three types.

- **Similar difference of relevance:** The RC network showed the highest relevance between community 1 and 2 and the lowest relevance between community 1 and 3. The differences between the values of community relevance were at almost the same level of approximately 0.2 (Table 1).
- **Low relevance except for one community pair:** The LFR network showed the highest relevance between communities 2 and 3 and low relevance between the other communities. Because communities 1 and 4, 1 and 5, as well as communities 4 and 5 in LFR network had no common neighbors, their degrees of community relevance were 0 (Table 2).
- **High relevance except for pairs with one community:** In the Tohoku network, community 1 had low relevance with all the other communities, which were strongly related. (Table 3).

**Table 1:** Relevance of community pairs in the RC network.

Pairs	Relevance
(1, 2)	**0.550**
(1, 3)	0.182
(2, 3)	0.350

Pairs represent the community pairs in the network and are indicated by their IDs.

**Table 2:** Relevance of community pairs in the LFR network.

Pairs	Relevance
(1, 2)	0.107
(1, 3)	0.046
(1, 4)	0.0
(1, 5)	0.0
(2, 3)	**0.471**
(2, 4)	0.225
(2, 5)	0.081
(3, 4)	0.153
(3, 5)	0.032
(4, 5)	0.0

Pairs represent the community pairs in the network and are indicated by their IDs.

**Table 3:** Relevance of community pairs in the Tohoku network.

Pairs	Relevance
(1, 2)	0.149
(1, 3)	0.211
(1, 4)	0.104
(1, 5)	0.075
(2, 3)	**0.570**
(2, 4)	**0.555**
(2, 5)	**0.394**
(3, 4)	**0.580**
(3, 5)	**0.449**
(4, 5)	**0.495**

Pairs represent the community pairs in the network and are indicated by their IDs.

### Visualization of the networks ranked by six node indexes

To verify the performance of the proposed index, we visualized the three types of networks by six node indexes: within-cluster degree Z (Z), Katz centrality (Katz), betweenness centrality (Bet), participation coefficient P (P), weighted P (PW), and inverse of Burt’s constraint (iBurt). In addition to the above-described differences in the community relevance, the networks showed different degree distributions, as characterized by a nearly uniform number of links (RC network), and diverse degrees (LFR and Tohoku network)(see Methods for details). We used a conventional force-directed placement method^33^ for the visualization. Because this method draws connected nodes closer to each other, the distance between visualized communities can be assumed as the extent of community relevance calculated based on common neighbors.

**Figure 3:**
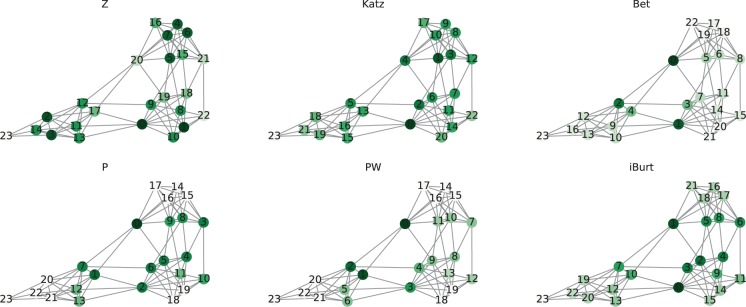
Index values of nodes in the RC network. Each network has 24 nodes and 84 edges with a rewiring probability of 0.2. The network had three communities named c1, c2, and c3 from the right to left. The color of the node represents the value of each index: Z, Katz, Bet, P, PW, and iBurt. The darker the color, the larger is the index value. The numbers on the nodes are their ranks in each index. The values of nodes can be found as Supplementary Table S2.

**Figure 4:**
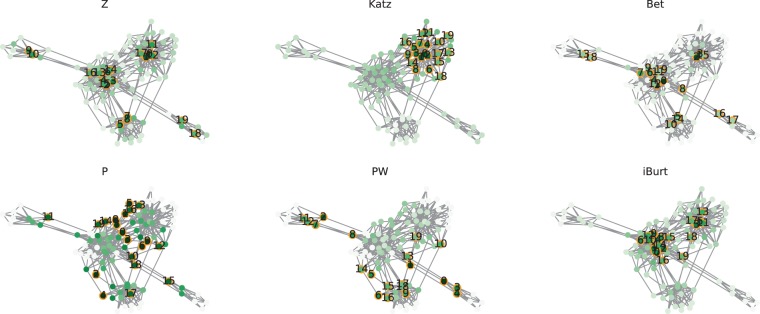
Index values of nodes in the LFR network. Each network has 100 nodes colored by their index values, 391 edges, and five communities with a mixing rate of 0.1. Top 20 nodes are marked by orange circles with labels of the node ranking in each network. The values of the top 20 nodes can be found in Supplementary Table S3.

**Figure 5:**
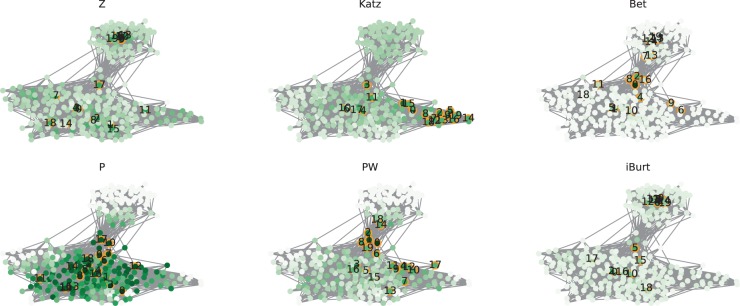
Index values of nodes in the Tohoku network. Each network has 217 nodes colored by their index values, 2696 edges, and five communities. Top 20 nodes are marked by orange circles with labels of the node ranking in each network. The upper community had fewer inter-community links compared to the other four communities below. The values of the top 20 nodes can be found in Supplementary Table S4.

In every visualization of the network, PW was the best at identifying the nodes connecting distant communities (Figs. 3, 4 and 5). Although P identified the nodes connecting plural communities, it did not distinguish the degree of differences between the communities because the P index evaluates only the degree of link dispersion at the community level. The other four indexes, namely Z, Katz, Bet, and iBurt, mostly depended on the number of links and did not identify community differences, especially when the network consists of nodes with diverse degrees.

#### RC network: uniform link density with different values of community relevance

In this experiment, the RC network had three communities with IDs c1, c2, and c3 from the right to left in Fig. 3. We named the only node that connects all three communities as “connector” and the node with the largest number of links as “hub.” We first examined how the six indexes evaluate these two typical nodes by comparing the rankings of the indexes. The Z index ranked the connector lowest at 20th, contrary to the other indexes, which ranked the connector quite highly. Bet, P, and PW gave the highest rank of 0th to the connector. Katz and iBurt also ranked it highly at 4th and 1st, respectively. On the other hand, the hub was given high ranks by all indexes, although P and PW ranked it lower at 2nd and 3rd, respectively. Z, Katz, and iBurt gave the highest rank of 0th to the hub, and Bet ranked it the second highest, reflecting that these indexes evaluate the number of links by definition (see Methods).

Next, we investigated if the six indexes could represent the community relevance. Except for Z, every index ranked gave higher ranks to nodes between communities compared with those inside communities. However, P and iBurt failed to capture the differences between communities because there is almost no difference in the order of the nodes connecting communities. For example, consider the top 4 nodes in the middle-positioned community c2. When ranked by P, the nodes connecting c1 and c3, which have low relevance, were ranked 2nd and 6th, respectively, and the nodes connecting c1 and c2, which have high relevance, were ranked 5th and 4th, respectively. Likewise, when ranked by iBurt, the nodes connecting communities with low relevance were ranked 0th and 3rd, and the nodes connecting communities with high relevance were ranked 2nd and 4th. On the other hand, Katz, Bet, and PW ranked nodes connecting distant communities higher, and PW was the best at ranking them highly. Katz did not evaluate nodes connecting distant communities, ranking the nodes as 16th, 15th, 13th, and 5th in c3. Bet also failed to rank nodes in c1 because it evaluated the nodes connecting close communities as 5th, 6th, and 8th. Overall, PW evaluated nodes properly in every community, rating the nodes connecting distant communities higher and those connecting close communities lower (Fig. 3).

#### LFR network: diverse link density with low community relevance

A notable point of P and PW is that they are not affected by the degrees, because they reflect the proportion of inter-community links to the total number of links. Therefore, P and PW are different from other network centrality indexes such as Bet and iBurt, which reflect the degrees by their definitions (see Methods). In other words, nodes can be ranked high in P or PW even if they have a small number of links, which is impossible for Bet and iBurt. To illustrate this characteristic, we generated an LFR network with diverse degrees holding five communities. In this network, only one community pair was strongly connected, while the others were weakly connected (Table 2, Fig. 4).

In this experiment, iBurt did not identify nodes connecting communities, although it has been considered to inversely represent structural holes in a network. In fact, except for P and PW, none of the indexes identified these connector nodes. The indexes Z, Katz, Bet, and iBurt ranked the nodes within communities highly, while P and PW ranked the nodes between communities highly. However, while the P index evenly evaluated nodes if they connected the communities, PW ranked the nodes connecting the most relevant communities lower than those mediating distant communities’ relationships. In short, only PW could detect nodes connecting distant communities in this network. (Fig. 4).

#### Tohoku network: diverse link density with high community relevance

The limit of the P appeared markedly in the Tohoku network with diverse degrees holding five communities, and we found that PW was the best at identifying the nodes connecting communities with low relevance. In this network, one community had relatively few inter-community links, while the others were strongly connected to each other (Table 3). Z, Katz, P, and iBurt highly ranked the nodes within these highly relevant communities and did not identify structural holes. On the other hand, both Bets and PW highly ranked the nodes between weakly connected communities, but PW showed better performance in identifying them (Fig. 5).

### Node rankings and attack tolerance of the network

The selection and removal of a few nodes that play a vital role in maintaining the network’s connectivity weaken the tolerance of inhomogeneous networks^34^. Here, we demonstrate that attack tolerance is also influenced by the structure of communities in the networks. We examined the attack targets that change network connectivity most and found that, among several node indexes, PW was the best at detecting the most influential nodes with a smaller number of edges in a heterogeneous network.

#### Changes in diameter by removed nodes and edges

**Figure 6:**
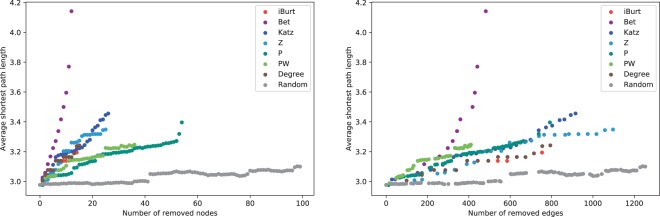
Changes in the diameter after the attack. The vertical axis represents the average shortest path length of the Tohoku network after the attack, and the horizontal axis is the cumulative number of the nodes (left figure) and the edges (right figure) that was removed after each attack. At every attack, we re-calculated the node values and we stopped the attack when the network was divided from the largest component.

We assumed that the absence of a node connecting distant clusters increases the diameter more than the absence of nodes connecting close clusters because the absence of a node connecting distant clusters can eliminate important links that many paths have to pass through to connect nodes in the network. We also noticed that attack tolerance comes at a high price in the case of target nodes with many links, such as influencers with many followers in social networks, because it is costly to remove or control them. Therefore, considering the cost of the removal, we investigated the number of removed edges as well as that of removed nodes (Fig. 6).

The inter-connectedness of a network is described by its diameter, which is defined as the average length of the shortest paths between any two nodes in the network. To address the attack tolerance of networks, we measured the diameter after sequential attacks on the top 15 nodes ranked by seven indexes: Z, Katz, Bet, P, PW, iBurt, and degree centrality in the Tohoku network. The Tohoku network has a scale-free property with degree distributions from 10 to 2,000 (see data in Methods). We compared the increasing patterns of the diameter after the sequential attacks on the nodes ordered by each index. We also included a random selection of 100 nodes in the comparison. As with the previous studies on inhomogeneous networks^34^, the diameter remains unchanged under an increasing level of errors by random selection. When the top-ranked nodes in any of seven indexes are eliminated, the diameter of the network increases faster than in the case of random selections. As expected, Bet was the best at finding the most influential nodes to shorten the diameter because it evaluates the number of links via the node. However, when we count the number of edges, PW was the best at detecting the most influential nodes with a smaller number of edges, which implies a low cost for the removal.

#### Rank correlation in networks with different numbers of communities

**Figure 7:**
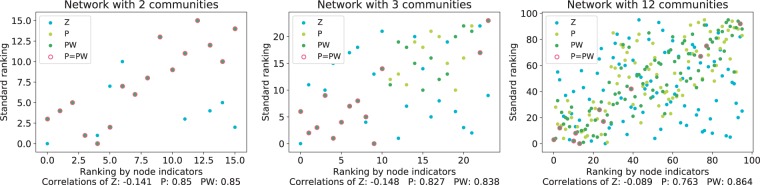
Rank correlations by path length and node indicators. The vertical axis represents the standard node ranking sorted by the average shortest path length after the attack, and the horizontal axis represents the node ranking ordered by the indexes Z, P and PW. Nodes with the same rankings in P and PW are indicated by red circles around green dots. We used RC networks with 2, 3, and 12 communities having 16, 24, and 96 nodes and 56, 84, and 336 edges, respectively, from left to right, with the Spearman’s rank correlations between standard node rankings and the rankings by the indexes.

To address the node values in the network, we extended and applied the experiment of attack tolerance in three steps. First, we measured the average shortest path length of the network when a node is removed and stored the node IDs in the increasing order of path length as the standard ranking. Second, we obtained the node rankings with three node indexes that discriminate communities in the network: Z, P, and PW. Third, we calculated the difference of the node ranking of each index with the standard ranking using Spearman’s rank-order correlation coefficient. By investigating the RC network with 2, 3, and 12 communities, we found that the rankings of PW show higher correlations with the standard rankings compared to the rankings of P, and the rankings of Z show no correlation (Fig. 7).

The rankings of P and PW were completely overlapped when the number of communities was two because there was only one community pair and the weight of P was constant. On the other hand, we detected differences between the rankings of P and PW in the network with three or more communities. When the network had three communities, middle-ranked nodes had different orders because there were three community pairs with different relevance values. However, at the lower ranks, the orders of P and PW overlapped with no correlation because low-ranked nodes had only within-community links and the same P and PW values of 0, which makes the ranking meaningless. The orders of P and PW were the same or close at the higher ranks, which might be because these nodes have links to all three communities. When the number of communities was 12, the rankings were different in general, and PW showed a higher correlation than P. In this case, as the ratio of inter-community links was relatively high, nodes that had only within-community links or had links to all 12 communities were very few. Therefore, the orders of P and PW did not match in almost every ranking in this network.

#### Rank correlation in networks with varied mixing rate

We generated six LFR networks with different inter-community link ratios (mu); in each network, we calculated Spearman’s rank correlation between the ranking ordered by each index and the average shortest path length after the attack. In the LFR network, the rank correlation decreased as mu increased because both P and PW evaluate links between communities (Table 4). However, PW showed the highest correlations in all networks, suggesting that PW identifies nodes that would make the average shortest path longer if they are removed. Typically, when the mixing parameter was 0.5 or 0.6, both P and PW have no correlation with the standard ranking, because the number of inter-community links exceeded that of within-community links and the community structure disappeared. We executed similar calculations in the RC networks with different numbers and sizes of communities, which can be found in Supplementary Tables S5 and S6. The analysis result of these RC networks showed a tendency in which PW has the highest correlations, suggesting its discriminating capacity on larger networks.

**Table 4:** Rank correlation in LFR networks with different link rates.

mu	Z	P	PW
0.1	−0.424	0.617	**0.895**
0.2	−0.569	0.596	**0.838**
0.3	−0.435	0.549	**0.766**
0.4	−0.357	0.404	**0.504**
0.5	0.019	0.258	**0.280**
0.6	−0.021	0.082	**0.163**

## Discussion

In this study, we proposed a new nodal index based on community structures and verified its performance. We designed the index PW using community relevance as the weight of the index P, which reflects the community coverage. To clarify the difference between P and PW, we used combined ego networks in Facebook. We showed that PW had a wider range than P, suggesting that it has a higher capacity to discriminate nodes in the network. In fact, closer inspection of ego networks revealed that PW showed better performance in detecting common friends than P. Then, to determine the capability of community discrimination of PW, we prepared three types of heterogeneous networks with different community relevance values and link densities. The visualization of these networks with the node ranking based on six indexes confirmed that PW is the best at identifying the nodes connecting distant communities. We also examined the attack tolerance of the networks by sequentially removing nodes ranked by each of the six indexes, as well as a simple degree ranking method and a random selection of nodes. We found that PW detects the most influential nodes with a smaller number of edges compared to the other indexes in a heterogeneous network. We generated networks with different numbers of communities and rewiring ratios. By analyzing rank-order correlation with the diameters of the networks, we revealed that PW showed a consistently higher correlation than P, suggesting that PW contributed to the improvement of identification of rare nodes that would make the diameter longer if they are removed. Considering the cost of removing nodes with many links, it would be useful to identify nodes that have fewer links but substantial impacts on the network, which can be detected using the proposed index.

A potential limitation of the study is its focus on the comparison between P and PW in the limited types of networks. Although we detected some differences with the other indexes such as iBurt, Bet, and Katz by the visualization of the networks and the changes in the diameter after the attack, further investigations are needed to clarify the characteristics of the proposed index, regarding the roles of the nodes in the heterogeneous networks. For designing the weight of the index P, we referred to information entropy^35^, considering the average of linkage probability related to the distances between communities. Finer-grained weighting schemes are expected for future research, such as random-walk based modular decomposition of Markov chain^36^, which incorporates the information as flow on network links^37^. Our approach to evaluate nodes by quantifying the local community structure can easily be applied to any network with diverse communities. In addition to finding key factors that implement shortcuts in small world networks, weak ties or bridges over structural holes, the proposed index could be applied to investigate the fusion of knowledge in different fields. It may also be used to plan strategies to mitigate phenomena related to the intensification of similar information, such as echo-chambers^38^ or filter bubbles^25,39^, by finding mediators to bring heterogeneity into the network.

## Methods

### Data

To verify the performance of the proposed index, we used two benchmark networks with different numbers of communities and rates of rewiring, as well as an inter-firm transaction network in Tohoku region based on real data (for visualization of the clusters in each network, see Supplementary Fig. S4).

#### Facebook network

The Facebook network was provided by SNAP^30^, which contained ego networks of 10 anonymous users that had the users’ friends lists^31^. In addition to the combined network with all users’ ego networks distributed by SNAP, we developed a new combined network with three users’ ego networks of G0, G2, and G6, for comparison.

#### RC network

The RC network has small world characteristics in that it simultaneously satisfies a small L and large C as well as a moderate average number of degrees^6^. First, we generated and visualized a simple network with the number of communities, number of nodes in a community, rewiring probability, and random seed set to 3, 8, 0.2, and 16, respectively, resulting in different community relevance values in the network. Next, for the experiments on attack tolerance, we generated three RC networks with 2, 3, and 12 communities; the number of nodes in each community was 8, and the rewiring probability was 0.2.

#### LFR network

The LFR network allows flexible and fine-grained parameter setting close to that of a real network^32^. We generated and visualized a network with the number of nodes, average number of degrees, maximum number of degrees, inter-community link ratio, and number of nodes in the communities set to 100, 8, 30, 0.1, and 5–50, respectively, resulting in different community relevance values with a scale-free property in the network. For the experiments on attack tolerance, we generated six LFR networks with 100 nodes, 8 degrees per node, and 10 communities with inter-community link ratios of 0.1–0.6. Note that networks lose community structure with a rewiring rate greater than 0.5, which disables the discriminating power of the indexes Z, P, and PW.

#### Tohoku network

The Tohoku network was generated using data provided by TOKYO SHOKO RESEARCH, LTD. (TSR), which consisted of annual data of firms and their business transactions with up to 20 business partners for each firm. The Tohoku network was generated with real inter-firm transaction data related to the Tohoku region in Japan in 2016, and the total volume of collected transaction data was 154,918. To avoid extreme deviation of the degree distribution, we excluded nodes with more than 2000 edges as well as those with less than 10 edges, resulting in 317 firms and 2412 transactions. As shown in Fig. 5, the Tohoku network had a community with fewer inter-community links and four tightly connected communities.

### Node Indicators based on centrality, neighbors, and link structures

Betweenness centrality^10,40^ measures the degree to which a node falls on the shortest path between other nodes in the network, and it is defined as follows:

3 $$Be{t}_{v}=\sum _{s,t\in V}\frac{\sigma (s,t|v)}{\sigma (s,t)},$$

where $v$ is the set of nodes, $\sigma (s,t)$ is the number of shortest $(s,t)$-paths, and $\sigma (s,t|v)$ is the number of paths passing through some node $v$ other than $s,t$. If $s=t,\sigma (s,t)=1$, and if $v\in s,t,\sigma (s,t|v)=0$.

Katz centrality^11^ takes into account the total number of paths between a pair of nodes. The Katz centrality ${x}_{i}$ of node $i$ is expressed as follows:

4 $${x}_{i}=\alpha \,\sum _{j}\,{A}_{ij}{x}_{j}+{\beta }_{i},$$

where A is the adjacency matrix of the graph with eigenvalues $\lambda$. The parameters $\alpha$ and $\beta$ are constants, where $\alpha  < \frac{1}{{\lambda }_{max}}$ and $\beta$ controls the initial centrality.

The measure to grasp structural holes was proposed as the opposite concept of network constraint, which represents the absence of a structural hole^13^. The sum of the local constraints of all pairs in a network for node $i$ is called Burt’s constraint, which is expressed as follows:

5 $$Bur{t}_{i}=\sum _{j\in {V}_{i},j\ne i}\,{({p}_{ij}+\sum _{q\in {V}_{i},q\ne i,j}{p}_{iq}{p}_{qj})}^{2},$$

where ${V}_{i}$ is the set of neighbors of $i$ and ${p}_{ij}$ is the normalized mutual weight of the edges joining $i$ and $j$, for each vertex $i$ and $j$. The mutual weight of $i$ and $j$ is the sum of the weights of edges joining them. Note that edge weights are assumed to be one if the graph is unweighted. By definition, Burt’s constraint does not count nodes more than three paths away from node $i$.

The role of each node is determined by its within-cluster degree and its participation coefficient P, which is defined by Eq. (1). The within-cluster degree and P characterize how the node is positioned in its own cluster and between clusters^15^. These two properties can easily be calculated after dividing the network into clusters. The within-cluster degree ${Z}_{i}$ measures how “well connected” node i is to other nodes in the cluster, and it is defined as follows:

6 $${Z}_{i}=\frac{{K}_{i}-{\bar{K}}_{Si}}{{\sigma }_{{K}_{{S}_{i}}}},$$

where ${K}_{i}$ is the number of links of node $i$ to other nodes in its community ${S}_{i}$, ${\bar{K}}_{{S}_{i}}$ is the average of K overall nodes in ${S}_{i}$, and ${\sigma }_{{K}_{{S}_{i}}}$ is the standard deviation of $K$ in ${S}_{i}$. ${Z}_{i}$ is high if the within-cluster degree is high and vice versa.

### Link prediction using community structure information

To extract the community structure of a network, we applied the fast and accurate Louvain method^29^, which is among several widely used modularity-based community division methods^41,42^. Then, we calculated the CRJC between communities, which showed the highest accuracy in predicting missing links in previous research^24^. CRJC measures the probability that both ${c}_{i}$ and ${c}_{j}$ have common neighbors, and it is defined as follows:

7 $${\rm{CRJC}}\,({c}_{i},{c}_{j})=\frac{|(\Gamma ({c}_{i})\cup V({c}_{i}))\cap (\Gamma ({c}_{j})\cup V({c}_{j}))|}{|(\Gamma ({c}_{i})\cup V({c}_{i}))\cup (\Gamma ({c}_{j})\cup V({c}_{j}))|},$$

where ${c}_{i}$ and ${c}_{j}$ are a pair of nodes within any one of the communities in the network defined by $C=\{{c}_{1},{c}_{2},\ldots ,{c}_{m}\}$. $V({c}_{i})=\{v|v\in V,{c}_{i}\in C(v)\}$ is the sum of the nodes in community ${c}_{i}$. $V$ represents the set of nodes in the network, and $C(v)$ is the set of communities holding node $v$. Γ$({c}_{i})$ indicates the neighbors of community ${c}_{i}$.

## Supplementary information

Supplementary Information.

## Data Availability

The datasets used in this article are all publicly available and cited in the references except for Tohoku network data provided by TOKYO SHOKO RESEARCH, LTD. (TSR).
