# Supplementary material for: Evaluating Nodes of Latent Mediators in Heterogeneous Communities
Source: Sci Rep. 2020 May 21;10:8456. doi: 10.1038/s41598-020-64548-6 (PMC7242394; doi:10.1038/s41598-020-64548-6)
Supplement: Supplementary file 1 — Supplementary Information. [file 41598_2020_64548_MOESM1_ESM.pdf]

Evaluating Nodes of Latent Mediators in Heterogeneous Communities

Hiroko Yamano, Kimitaka Asatani, Ichiro Sakata

Supplementary Information

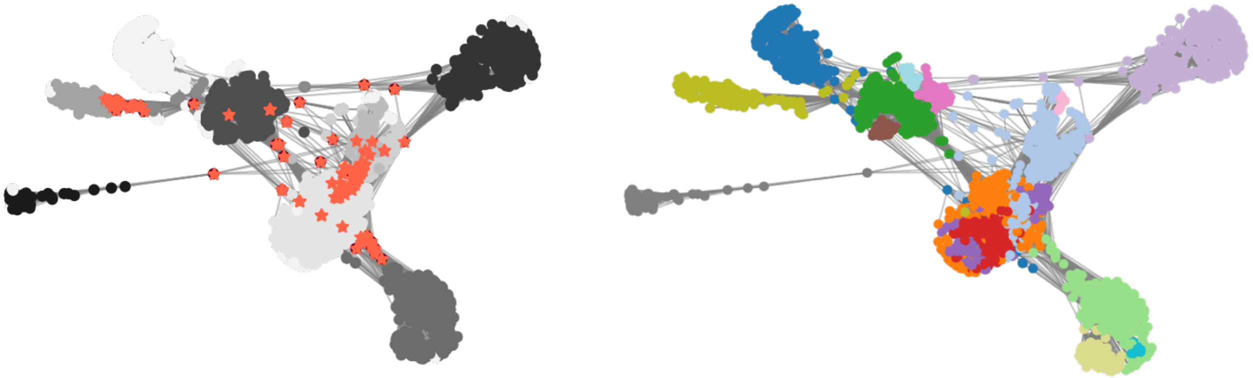

Figure S1. Facebook 10 ego users' network with 15 communities. The left and right graphs represent the same combined Facebook network with different node shapes and colors. Left: The star-shaped red nodes represent common friends between ego users. The thickness of gray nodes represents the corresponding user's friends. Right: The nodes are colored according to the community they belong to, calculated by the modularity-based algorithm of the Louvain method. We can see several users have plural communities. The link density between communities tended to higher within a user than between the users.

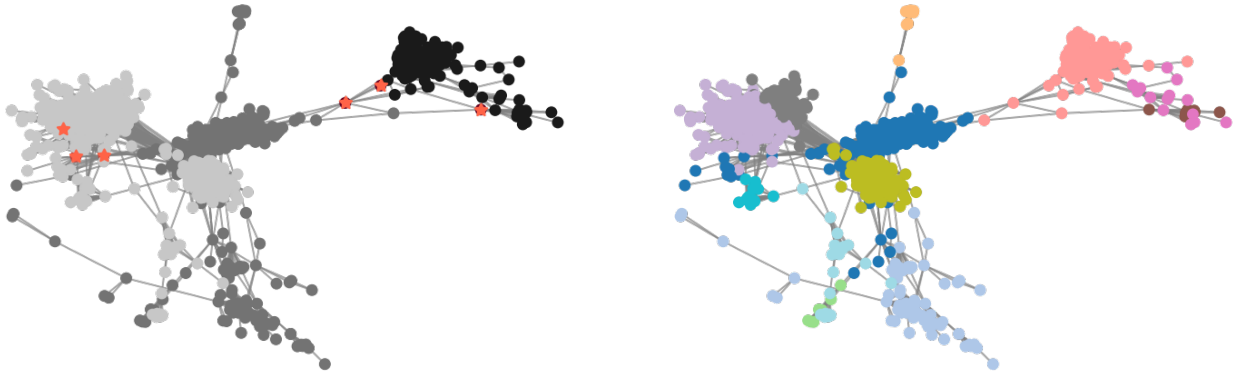

Figure S2. Facebook three ego users' network with 12 communities. Symbols and colors are as in Figure 8.

	All (10 users)				G026 (3 users)			
	P_n	P_v	PW_n	PW_v	P_n	P_v	PW_n	PW_v
0	2359	0.719	804	3.144	650	0.587	1307	2.565
1	641	0.691	2902	2.553	635	0.581	243	2.072
2	2007	0.667	3872	2.54	1141	0.579	296	2.001
3	2508	0.667	541	2.52	1249	0.568	295	1.907
4	2936	0.667	3562	2.298	1240	0.559	44	1.9
5	3244	0.667	891	2.269	1089	0.555	264	1.818
6	3583	0.661	921	2.198	935	0.551	1272	1.797
7	3081	0.658	1880	2.198	829	0.542	1195	1.797
8	3609	0.656	2130	2.198	1132	0.541	236	1.657
9	1050	0.643	776	2.19	44	0.539	225	1.556
10	2627	0.64	1859	2.186	1196	0.539	214	1.554
11	3833	0.64	145	2.129	1129	0.538	281	1.554
12	1897	0.639	377	2.088	940	0.536	533	1.546
13	3981	0.628	61	2.041	845	0.535	83	1.533
14	783	0.625	3081	1.865	1092	0.527	224	1.441
15	937	0.625	116	1.791	1073	0.526	521	1.374
16	1048	0.625	2394	1.772	549	0.524	310	1.373
17	1551	0.625	2814	1.772	1248	0.518	1083	1.317
18	2390	0.625	3469	1.693	602	0.516	249	1.248
19	3610	0.625	2038	1.649	814	0.514	374	1.192

Table S1. Node values in the Facebook networks with P and PW. The values of the top 20 nodes in the two kinds of Facebook networks are shown in the order of the node ranking calculated by the two indexes of P_v and PW_v with their node IDs of P_n and PW_n.

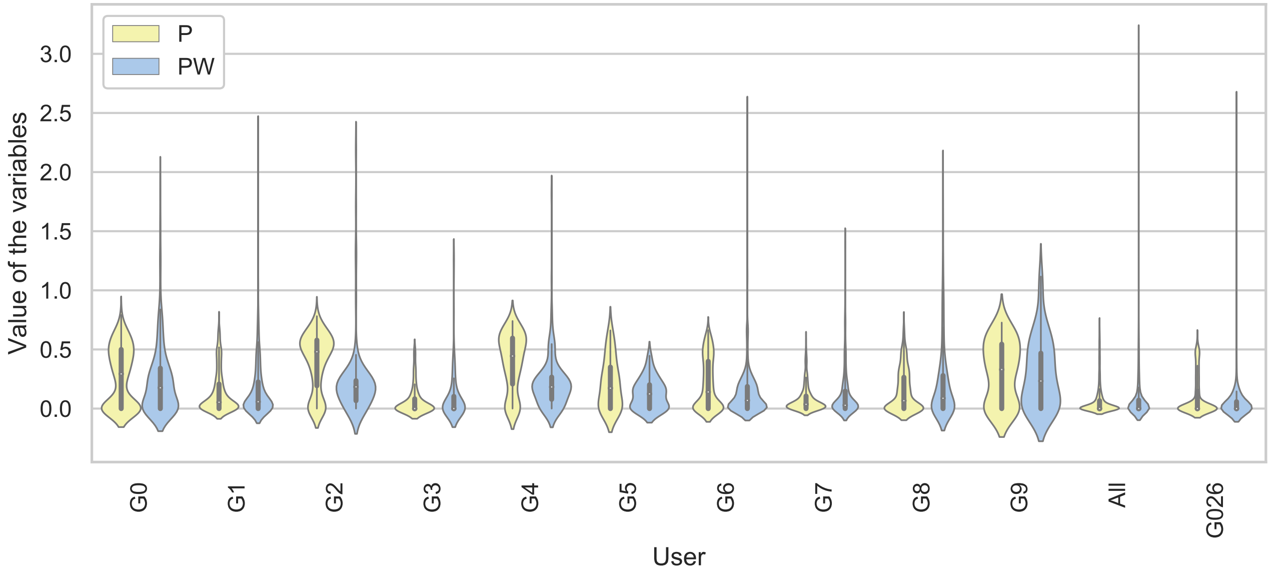

Figure S3. Comparison of the distributions of P and PW in Facebook networks. The violin plots represent the distributions of P and PW for each of 10 Facebook users (G0-G9), and two combined networks of all users and three users (G0, G2, and G6). The values of the X-axis and Y-axis are as in Figure 1.

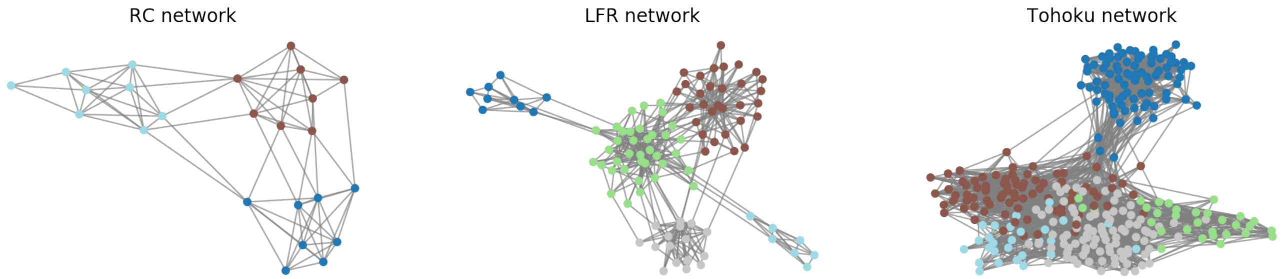

Figure S4. Three networks, colored by the communities. The RC network has three communities with modest differences in community relevance. The LFR network has five communities with low relevance except for one community pair that is tightly connected. The Tohoku network has five communities with high relevance except for pairs with one community that is loosely connected to the other four communities. Each network was clustered by the modularity-based algorithm of the Louvain method.

ID	Z	Katz	Bet	P	PW	Burt
0	0.905	0.208	0.007	0.000	-0.000	0.416
1	-0.302	0.227	0.046	0.375	0.224	0.310
2	0.905	0.246	0.059	0.346	0.207	0.293
3	-1.508	0.226	0.196	0.531	0.632	0.258
4	-1.508	0.204	0.039	0.408	0.244	0.295
5	0.905	0.208	0.007	0.000	-0.000	0.416
6	0.905	0.208	0.007	0.000	-0.000	0.416
7	-0.302	0.189	0.001	0.000	-0.000	0.436
8	0.258	0.207	0.025	0.245	0.146	0.324
9	0.258	0.229	0.091	0.406	0.324	0.283
10	-0.775	0.211	0.037	0.408	0.244	0.287
11	0.258	0.184	0.004	0.000	-0.000	0.405
12	1.291	0.258	0.178	0.420	0.441	0.249
13	1.291	0.204	0.006	0.000	-0.000	0.390
14	-0.775	0.213	0.044	0.408	0.244	0.275
15	-1.807	0.163	0.022	0.320	0.191	0.360
16	-0.626	0.204	0.087	0.449	0.595	0.328
17	0.209	0.198	0.037	0.245	0.257	0.389
18	1.043	0.185	0.031	0.000	-0.000	0.425
19	1.043	0.185	0.031	0.000	-0.000	0.425
20	0.209	0.216	0.157	0.406	0.538	0.308
21	0.209	0.198	0.037	0.245	0.257	0.389
22	0.209	0.166	0.020	0.000	-0.000	0.446
23	-2.294	0.111	0.000	0.000	-0.000	0.565

Table S2. Node values in the RC networks with six indexes. The values of all 24 nodes in the RC network are represented in the order of node ID calculated by the six indexes of Z, Kats, Bet, P, PW, and Burt. For the network visualization of node ranking, we used the order of inverse Burt, according to the definition of Burt's constraint and structural holes.

Rank	Z_n	Z_v	$Katz_n$	$Katz_v$	Bet_n	Bet_v	P_n	P_v	PW_n	PW_v	$Burt_n$	$Burt_v$
0	100	3.155	100	0.371	97	0.116	2	0.375	30	0.804	23	0.555
1	98	2.633	98	0.325	99	0.111	3	0.375	39	0.804	33	0.537
2	99	2.591	94	0.278	98	0.107	6	0.375	34	0.716	31	0.516
3	97	2.091	87	0.267	100	0.096	12	0.375	50	0.615	44	0.506
4	96	1.924	90	0.266	96	0.082	13	0.375	60	0.615	11	0.489
5	88	1.890	81	0.226	85	0.078	15	0.375	6	0.559	1	0.478
6	95	1.757	77	0.190	93	0.074	16	0.375	13	0.559	9	0.469
7	85	1.638	71	0.184	89	0.072	18	0.375	62	0.548	19	0.460
8	86	1.638	78	0.165	72	0.056	20	0.375	55	0.548	45	0.418
9	68	1.633	49	0.160	82	0.048	22	0.375	37	0.477	50	0.400
10	69	1.633	53	0.149	88	0.046	72	0.340	51	0.460	60	0.400
11	90	1.589	58	0.137	95	0.044	34	0.320	68	0.442	30	0.395
12	94	1.589	57	0.134	92	0.043	36	0.320	69	0.442	4	0.368
13	93	1.257	56	0.129	69	0.040	24	0.320	72	0.438	7	0.364
14	91	1.257	54	0.118	86	0.038	25	0.320	41	0.414	14	0.360
15	92	1.257	59	0.110	94	0.038	30	0.320	47	0.414	21	0.346
16	89	1.090	32	0.108	30	0.037	32	0.320	48	0.414	34	0.340
17	87	1.067	26	0.108	50	0.034	37	0.320	85	0.378	27	0.340
18	44	1.029	51	0.105	62	0.034	39	0.320	86	0.378	22	0.339
19	50	1.029	27	0.102	91	0.033	40	0.320	78	0.340	38	0.338

Table S3. Node values in the LFR networks with six indexes. The values of the top 20 nodes in the LFR network are shown in the order of the node ranking calculated by the six indexes of Z_v, $Katz_v$, Bet_v, P_v, PW_v, and $Burt_v$ with their node IDs of Z_n, $Katz_n$, Bet_n, P_n, PW_n, and $Burt_n$.

Rank	Z_n	Z_v	$Katz_n$	$Katz_v$	Bet_n	Bet_v	P_n	P_v	PW_n	PW_v	$Burt_n$	$Burt_v$
0	23	4.704	16	0.302	25	0.131	295	0.708	52	1.193	177	0.200
1	59	4.241	171	0.231	23	0.120	185	0.702	35	1.141	203	0.200
2	29	3.537	24	0.180	35	0.072	131	0.700	197	0.914	294	0.200
3	22	3.289	25	0.172	22	0.058	134	0.700	131	0.795	30	0.167
4	93	3.289	21	0.169	36	0.051	133	0.698	171	0.775	96	0.167
5	5	3.216	17	0.168	93	0.050	161	0.694	133	0.771	109	0.167
6	4	3.131	23	0.164	16	0.041	36	0.694	239	0.737	135	0.167
7	73	3.032	26	0.147	39	0.041	239	0.691	269	0.718	184	0.167
8	28	3.009	304	0.143	51	0.037	105	0.685	51	0.705	244	0.165
9	2	2.960	204	0.140	171	0.031	130	0.678	248	0.693	151	0.164
10	7	2.960	22	0.139	21	0.029	52	0.672	316	0.675	235	0.145
11	16	2.631	36	0.138	67	0.027	277	0.667	169	0.667	34	0.143
12	14	2.534	211	0.130	1	0.027	171	0.665	179	0.657	128	0.143
13	11	2.449	48	0.130	209	0.027	149	0.660	295	0.650	232	0.139
14	102	2.367	43	0.128	13	0.025	201	0.656	79	0.647	237	0.132
15	61	2.305	179	0.127	4	0.022	102	0.653	185	0.640	169	0.128
16	3	2.193	147	0.126	52	0.022	21	0.653	161	0.631	125	0.128
17	25	2.132	18	0.126	2	0.019	35	0.643	30	0.624	145	0.125
18	98	2.049	45	0.126	84	0.019	249	0.640	39	0.620	162	0.125
19	1	2.022	46	0.124	5	0.018	126	0.640	25	0.612	180	0.125

Table S4. Node values in the Tohoku networks with six indexes. The values of the top 20 nodes in the Tohoku network are shown in the order of the node ranking calculated by the six indexes of Z_v, $Katz_v$, Bet_v, P_v, PW_v, and $Burt_v$ with their node IDs of Z_n, $Katz_n$, Bet_n, P_n, PW_n, and $Burt_n$.

Cn	Z	P	PW
2	-0.141	**0.850**	**0.850**
3	-0.148	0.827	**0.838**
4	0.048	0.507	**0.733**
5	-0.174	0.676	**0.825**
6	-0.194	0.663	**0.823**
7	-0.208	0.792	**0.863**
8	0.006	0.727	**0.792**
9	-0.119	0.737	**0.864**
10	-0.068	0.785	**0.880**
11	-0.022	0.711	**0.846**
12	-0.089	0.763	**0.864**
13	-0.126	0.75	**0.852**
14	-0.157	0.746	**0.863**
15	-0.101	0.706	**0.861**
16	-0.074	0.779	**0.889**
17	-0.027	0.775	**0.869**

Table S5. Rank correlation in the RC networks with different numbers of communities. For the additional experiments on attack tolerance, we generated 16 RC networks with the different number of communities (Cn) of 2 to 17, 8 nodes per community, and the fixed probability of rewiring each edge of 0.2. In each network, we calculated Spearman's rank correlation between the ranking ordered by each index and the average shortest path length after the attack. PW showed the highest correlations in all networks, suggesting that PW identifies nodes that would make the average shortest path longer if they are removed. Typically, when the number of communities was two, the rank correlation of P and PW was the same, because there was only one community pair and the weight of P was constant.

Sn	Z	P	PW
5	-0.597	0.735	**0.749**
6	0.238	0.757	**0.871**
7	0.064	0.718	**0.871**
8	-0.174	0.676	**0.833**
9	-0.159	0.844	**0.888**
10	-0.066	0.869	**0.893**
11	0.071	0.847	**0.909**
12	-0.182	0.903	**0.93**
13	-0.284	**0.914**	0.912
14	-0.09	0.828	**0.859**
15	0.014	0.861	**0.894**
16	-0.187	0.886	**0.89**
17	-0.16	**0.857**	0.856
18	-0.21	0.842	**0.882**
19	-0.186	0.888	**0.903**
20	-0.129	0.881	**0.887**

Table S6. Rank correlation in the RC networks with different sizes of communities. For the additional experiments on attack tolerance, we generated 16 RC networks with different sizes of the community (Sn) with 5 to 20 nodes in each community, five communities, and the fixed probability of rewiring each edge of 0.2. In each network, we calculated Spearman's rank correlation between the ranking ordered by each index and the average shortest path length after the attack. PW tended to show higher correlations than Z and P.
